# CT-Based Micromotion Analysis After Locking Plate Fixation of AO Type C Distal Radius Fractures

**DOI:** 10.1007/s43465-023-01020-3

**Published:** 2023-11-03

**Authors:** Eva Lundqvist, Henrik Olivecrona, Per Wretenberg, Marcus Sagerfors

**Affiliations:** 1 Faculty of Medicine and Health, Örebro University, Örebro, Sweden; 2 Department of Orthopedics and Hand Surgery, Örebro University Hospital, Södra Grev Rosengatan, 70185 Örebro, Sweden; 3 Department of Molecular Medicine and Surgery, Karolinska Institute, Stockholm, Sweden

**Keywords:** Articular, Distal radius fractures, Computed tomography, Dorsal ulnar corner, Internal fixation, Micromotion analysis, Volar locking plate, Outcomes, Trauma, Wrist

## Abstract

**Background:**

Volar locking plate fixation (VLPF) is the most common method for operative fixation of distal radius fractures (DRF). The dorsal ulnar corner (DUC) can be difficult to stabilize as the fragment is small and not exposed when using the volar approach. The purpose of this study was to study fracture fragment migration after VLPF of AO type C DRF, using a volume registration technique of paired CT scans with special focus on the DUC fragment.

**Materials and Methods:**

This pilot study included ten patients with AO type C DRF, all operated with VLPF. The primary outcome was radiographic outcome. Postoperative and 1-year scans were compared and analyzed. Fragment migration was assessed with CT-based micromotion analysis (CTMA), a software technique used for volume registration of paired CT scans.

**Results:**

All plates were stable over time. Two patients showed signs of screw movement (0.2–0.35 mm and 0.35– > 1 mm respectively). Postoperative reduction was maintained, and there was no fragment migration at the 1-year follow-up except for one case with increased dorsal tilt. The DUC fragment was found in 8/10 cases, fixated in 7/8 cases, and not dislocated in any case at the 1-year follow-up.

**Conclusion:**

The CTMA results indicate that variable-angle VLPF after AO type C DRF can yield and maintain a highly stable reduction of the fracture fragments. The DUC fragment remained stable regardless of the number of screws through the fragment. CT volume registration can be a valuable tool in the detailed assessment of fracture fragment migration following volar plate fixation of DRFs.

## Introduction

Distal radius fractures (DRFs) are common, comprising 18% of all fractures among adults in an orthopedic trauma unit, and their incidence is increasing due to an aging population [1, 2]. There has been a shift during recent decades from non-operative and other operative treatments toward volar plate fixation aimed at restoring the anatomy and improving the clinical and radiographic outcome [3, 4]. The volar locking plate has shown good clinical and radiographic outcomes, even for AO type C fractures [5, 6]. It allows early mobilization, which is beneficial for early return of function. Known complications include tenosynovitis, tendon rupture, and median nerve irritation [7]. The frequency of hardware removal is 15–30% [7, 8]. However, concerns have been raised that a single volar locking plate may not be sufficient for complex intra-articular AO type C fractures involving the dorsal ulnar corner (DUC) [9, 10]. The DUC plays a critical role in the DRUJ, anchoring the dorsal distal radioulnar ligament as well as providing dorsal rim stability and preservation of appropriate dorsal tilt [9]. To prevent postoperative displacement of the fragment, stabilization with at least one screw through the volar locking plate has been proposed; however, the size of the DUC fragment is often small [10].

CT-based micromotion analysis (CTMA) is an image post-processing volume registration technique used for analyzing and measuring migration between two CT examinations [11]. The registration (i.e., bringing the images into spatial alignment) and calculation are based on the relative micromotion between two rigid bodies, such as non-deforming bone and the implant. The method has shown the clinically relevant precision comparable to radiostereometric analysis (RSA) [11–13]. RSA was introduced in 1974 and is considered the gold standard for assessment of implant migration [14]. However, it requires specialized equipment, trained staff, and strict patient positioning during the examination, and so new methods have been developed. The volume registration technique has been used to analyze motion between the scaphoid and the lunate during the dart-throwing motion, and to analyze triquetral motion after lunocapitate arthrodesis [15, 16], but to our knowledge has not been used to assess fracture fragment migration in DRFs.

In this study, we used CTMA for the first time in a clinical setting in patients surgically treated for AO type C DRF. The aim of this study was to evaluate Computed Tomography Micromotion Analysis (CTMA) in a clinical setting for follow-up of surgically treated AO type C distal radius fracture patients.

## Materials and Methods

This prospective study was conducted at the Department of Orthopedics and Hand Surgery, Örebro University Hospital, a tertiary referral center in Sweden. The study was approved by the Swedish Ethical Review Authority (EPM, 2019-04377). All patients gave written informed consent before participation, according to the Helsinki declaration [17]. The study was registered in the Swedish research database FoU in Sweden (www.researchweb.org/is/sverige, ref: 272589). The sample consisted of 10 adult patients with AO type C fractures treated with variable-angle volar locking plates (TriMed, Santa Clarita, CA, USA) between March 25th and October 28th 2020. The plate is made in stainless steel and allows for placement of 3–7 screws in the radius shaft (3.2 mm) and double rows of 2.3 mm locking screws distally with up to 30° optional angulation. Inclusion and exclusion criteria are presented in Table 1. All operations were performed by hand surgeons.

**Table 1:** Inclusion and exclusion criteria

Inclusion criteria	Exclusion criteria
Age 18–80 years	Previous fracture of the same wrist
Operation within 14 days from injury	Bilateral fractures
AO type C with one or more of the following:	Other concomitant fractures
> 20° dorsal angulation of the distal radial articular surface	Open fracture
> 2 mm ulnar plus	Fracture extending to the diaphysis
> 1 mm incongruence in the radiocarpal joint	Ongoing chemotherapy or radiotherapy
> 1 mm incongruence in the distal radioulnar joint	Metabolic diseases that affect bone
	Dementia
	Mental illness
	Alcohol or drug abuse
	Difficulty understanding Swedish
	Severe neurological disease
	Severe cardiopulmonary disease
	Associated injuries (e.g., ligament injury or other factures in hand/arm)

### Surgical Technique

**Fig. 1:**
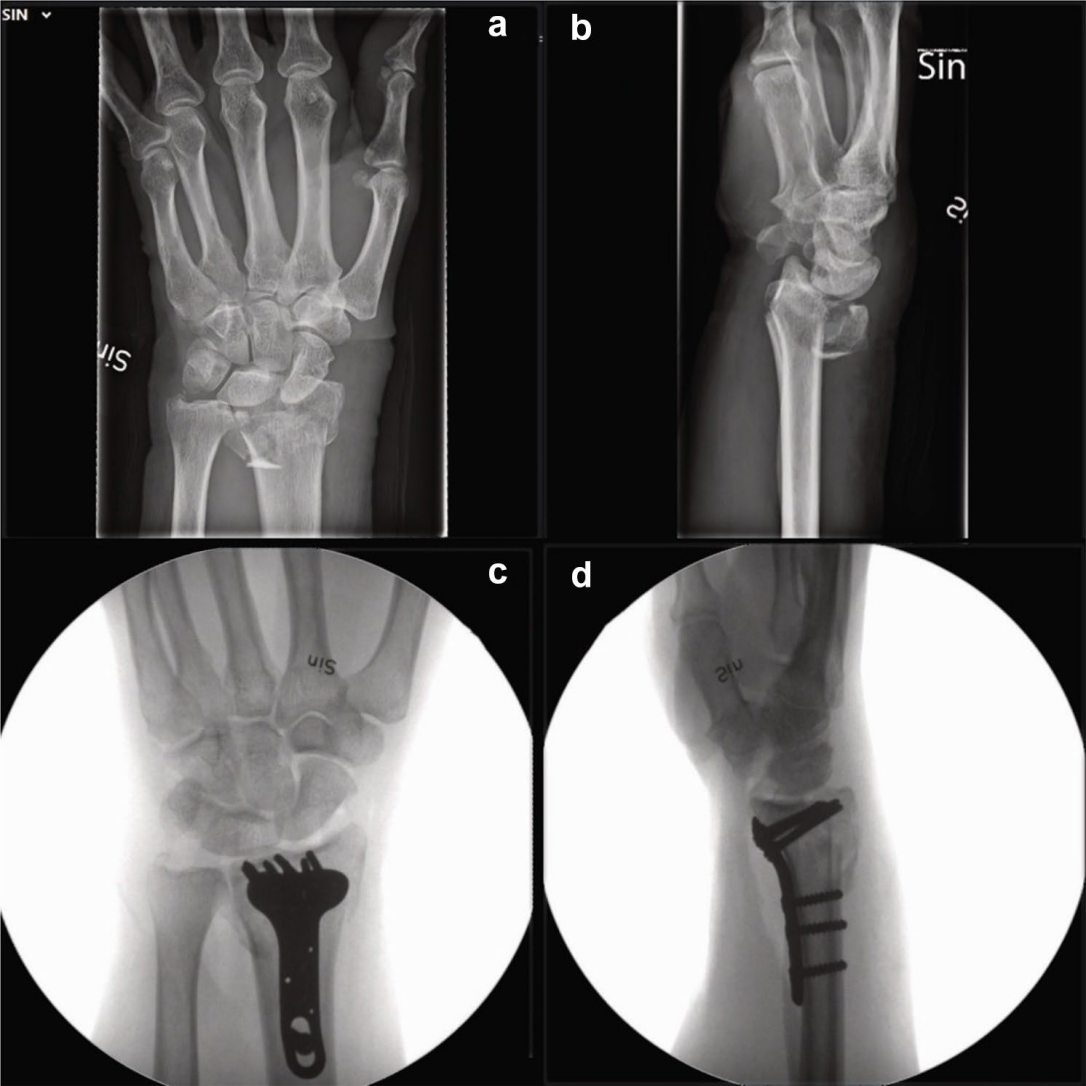
Pre- and intraoperative radiographs. Patient number 6

Surgery was performed according to department routine under general anesthesia with a brachial plexus block and tourniquet. A volar central incision was made to visualize the volar ulnar portion of the distal radius, and the carpal tunnel was opened through the same incision since the volar corner often is involved in AO type C fractures. The volar portion of the distal radius was exposed between the finger flexors ulnarly, and the median nerve and the thumb flexor radially. The pronator quadratus was divided with a central split. The central incision provides a good visualization of the volar lunate facet, also called the critical corner, and facilitates fixation of the DUC fragment [18]. The volar cortex was reduced, and the volar plate was placed (Fig. 1). The pronator quadratus was repaired using resorbable sutures if feasible.

All patients were seen by a hand therapist on the first day postoperatively for instructions regarding exercises to reduce edema and active finger range of motion exercises. After two weeks with a cast, an orthosis was used for an additional two weeks. Gentle mobilization was initiated two weeks postoperatively. The orthosis was removed during active wrist and finger range of motion exercises. After three months, clinical outcome measurements and radiographic evaluation were performed. There were no further load restrictions if the fracture was considered healed. Fracture healing is of today not properly defined and relies on different criteria including mechanical stability [19].

In case of an associated ulnar styloid fracture, the stability of the distal radioulnar joint (DRUJ) was evaluated intraoperatively after plate fixation of the DRF. If DRU instability was found, the styloid was fixated with a 2.0 mm locking ulna hook plate (DePuy Synthes, West Chester, PA, USA).

### Clinical Evaluation

At the 1-year follow-up, a hand therapist performed clinical measurements including wrist range of motion (ROM), hand grip strength, visual analog scale (VAS) pain scores, and patient-reported outcome measurements (PROMs).

Validated Swedish translated versions of the Patient-Rated Wrist Evaluation (PRWE) score and the short version of the Quick Disabilities of the Arm Shoulder and Hand (QuickDASH) questionnaire were used [20, 21]. The PRWE is a 15-item questionnaire with a maximum score of 100, where 0 represents no pain or disability in activities of daily living. The QuickDASH questionnaire evaluates a patient’s upper extremity disability during the last week. An eleven-item questionnaire is used to calculate a score ranging from 0 to 100, where 100 represents the most severe disability and symptoms.

Wrist ROM (flexion, extension, radial deviation, ulnar deviation, and pronation and supination (degrees)) was evaluated using a goniometer according to guidelines from the Swedish National Quality Registry for Hand Surgery [22].

Hand grip strength in kg was measured with a Jamar Hand Dynamometer (Biometrics Ltd, Newport, UK). The mean value of three measurements was calculated [22]. For right-handed patients, correction of grip strength was calculated as a percentage of the strength on the uninjured side according to the 10% rule [23].

Pain was evaluated both at rest and during activity using the VAS pain score (0 = no pain, 10 = worst imaginable pain).

### Radiographic Evaluation and Motion Analysis

The AO classification of the DRFs was assessed by the operating surgeon using preoperative radiographs and intraoperative findings.

CT scan examination was performed preoperatively, postoperatively within 2 days after surgery, and 1 year postoperatively. Double examinations were performed on the first five patients at the postoperative scans, to assess the reliability of the method. The data volumes were acquired according to the protocol used for standard imaging of the wrist (Somatom Definition Flash, Siemens Healthineers, Germany, Erlangen/Forchheim. kV 100, Effective mAs 100, slice thickness/overlap 0.6/0.4 mm, Kernel Br58).

Batra radiographic score was calculated and assessed on postoperative radiographic examinations (anteroposterior and lateral views). The measurements were performed by a single hand surgeon. This score includes radial angle, radial length, volar tilt, and articular incongruency and congruency of the DRUJ. The parameters were summarized and graded in four categories: excellent (90–100), good (80–89), fair (70–79), or poor (< 70) [24].

Postoperative and 1-year follow-up CT examinations were assessed and analyzed. Presence of a DUC fragment was assessed, and the number of screws in each DUC fragment was measured, as was the length of the screws in relation to the distance between the volar and dorsal cortex of the distal radius at the sigmoid notch. Finally, articular incongruence (step off or gap formation) was assessed.

Paired CT volumes were analyzed using the image post-processing volume registration tool CTMA (Sectra CTMA, Sectra, Linköping, Sweden). This tool provides a method for graphically visualizing and numerically calculating the motion in space between two rigid bodies based on CT volume registration [19]. These can be non-deforming bone or an implant. In our setting, the volar plate and the radial shaft proximal to the fracture can be considered rigid bodies, but the individual distal fracture fragments cannot, since remodeling is expected to occur.

First the double examinations were studied, and then the postoperative examination was registered to the 1-year examination. The plate was registered (brought into spatial alignment), and the appearance of the radius proximal to the fracture was examined in 3D and 2D images. Thereafter, the radius proximal to the fracture was registered. Two points, one proximal and one distal on the plate, were chosen as measurement points of the plate relative to the radius. The system by default also gives the movement at a center of mass point. Thereafter the movements of the distal fracture fragments were visualized in multiplanar reconstruction overlay images aligned along the long axis of the radius.

Movement of the screws relative to the plate was analyzed by the CTMA software, using color mapping (intervals: < 0.2 mm, 0.2–0.35 mm, > 0.35 mm).

## Results

All 10 patients completed the 1-year follow-up. Demographic characteristics are presented in Table 2. The ulnar styloid was operatively stabilized in one patient. Clinical outcome measures are presented in Table 3.

**Table 2:** Demographic data

Male/female	Age, median (range)	Side fractured, right/left	Hand dominance, right/left	DRF AO type, A/B/C
3/7	54.5 (20–63)	2/8	9/1	0/0/10

**Table 3:** Outcome 1 year postoperatively

Outcome measure	Injured side, median (range)	Percentage of uninjured side, median (range)
Pronation (degrees)	75 (70–85)	94 (82–108)
Supination (degrees)	80 (65–90)	100 (76–117)
Dorsal extension (degrees)	55 (35–75)	89 (54–115)
Volar flexion (degrees)	70 (45–85)	80 (64–100)
Radial deviation (degrees)	25 (20–30)	100 (80–167)
Ulnar deviation (degrees)	35 (20–50)	100 (63–133)
Grip strength (kg)	23.9 (13.1–57.5)	76 (65–104)
Grip strength (kg), corrected		83 (66–116)
VAS at rest (cm)	0.0 (0.0–0.0)	
VAS during activity (cm)	0.1 (0.0–2.5)	
PRWE (points)	4.0 (0.0–11.0)	
QuickDASH (points)	2.3 (0.0–13.6)	

**Fig. 2:**
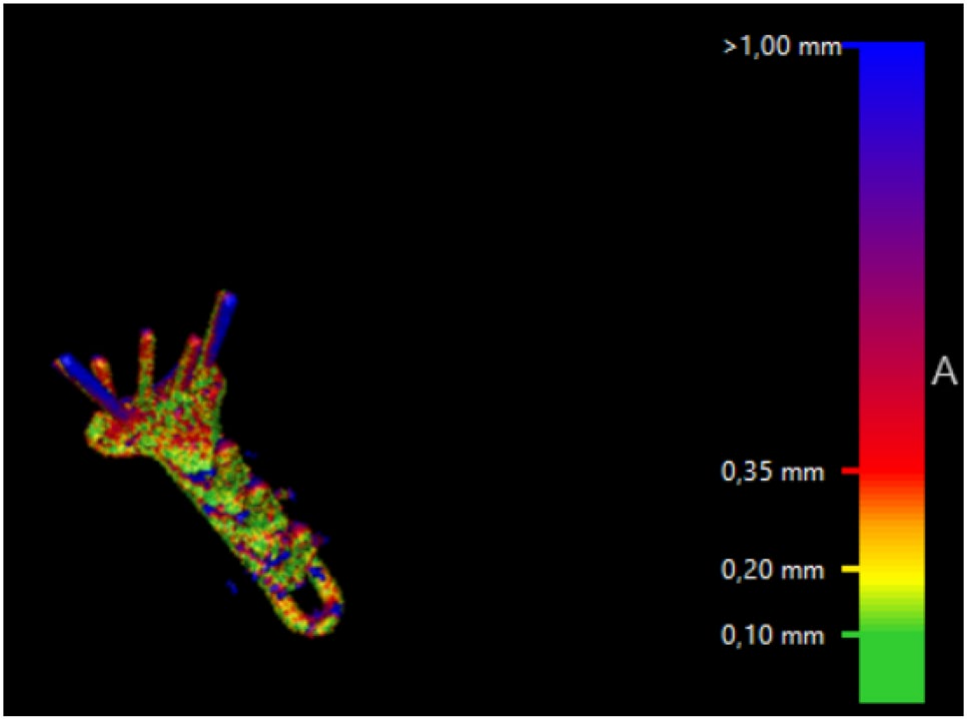
Alignment result with color mapping. Patient no. 8 with screw migration

**Fig. 3:**
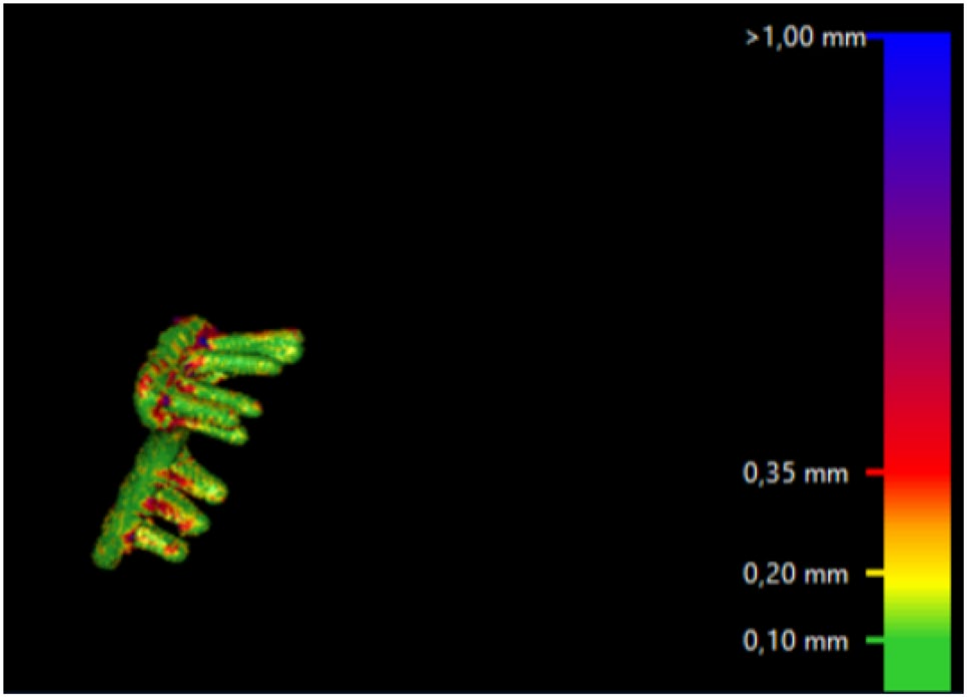
Alignment result with color mapping. Patient no. 2 without screw migration

Registration of the plate showed excellent (< 0.2 mm) overlapping of the surface of the plate. There were no signs of plate deformity over time. After registration of the plate, the radius proximal to the fracture was also aligned, indicating that all plates were stable over time. However, two patients (nos. 8 and 10) showed signs of screw movement (Figs. 2, 3).

**Fig. 4:**
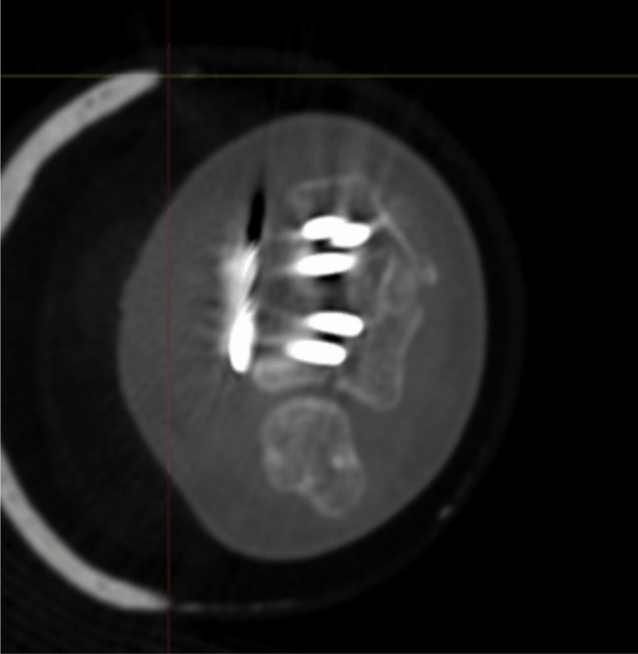
Postoperative scan of patient no. 2, with DUC fragment outlined

**Fig. 5:**
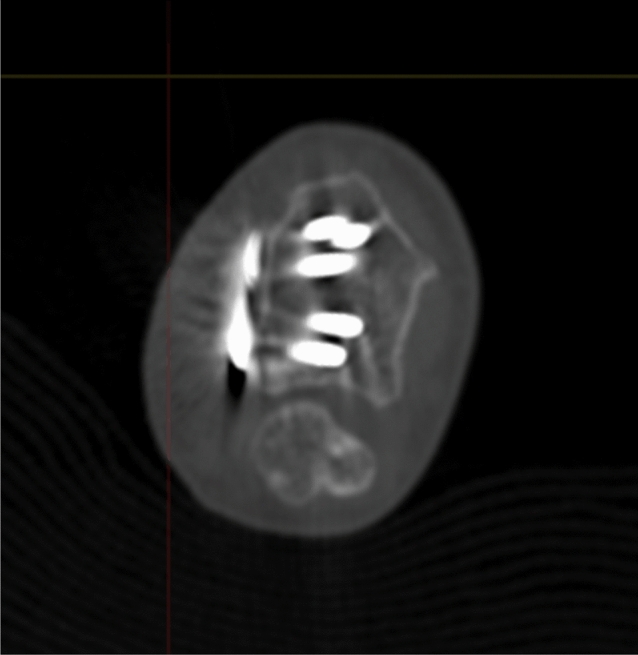
One-year follow-up of patient no. 2, with no migration/dislocation of the DUC fragment

**Fig. 6:**
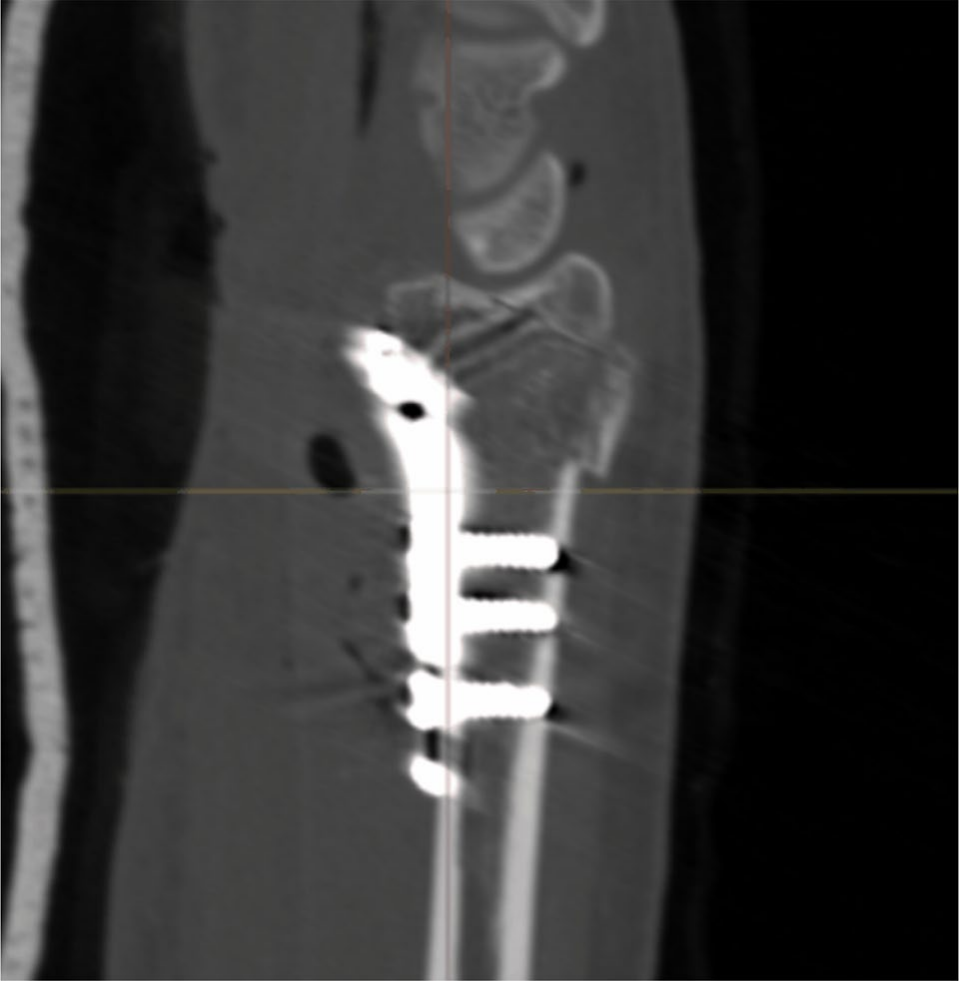
Postoperative scan of patient no. 2, with a dorsal fragment

**Fig. 7:**
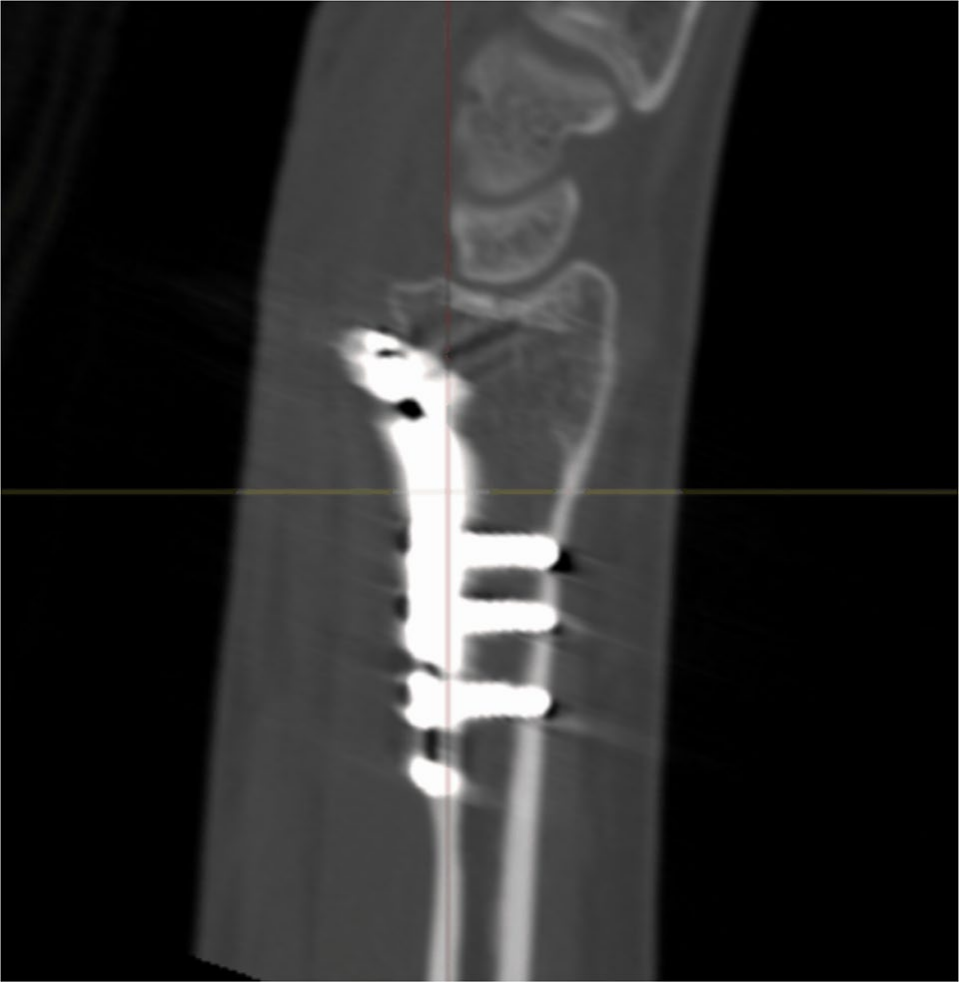
One-year follow-up of patient no. 2, with remodulation of the dorsal cortex

**Fig. 8:**
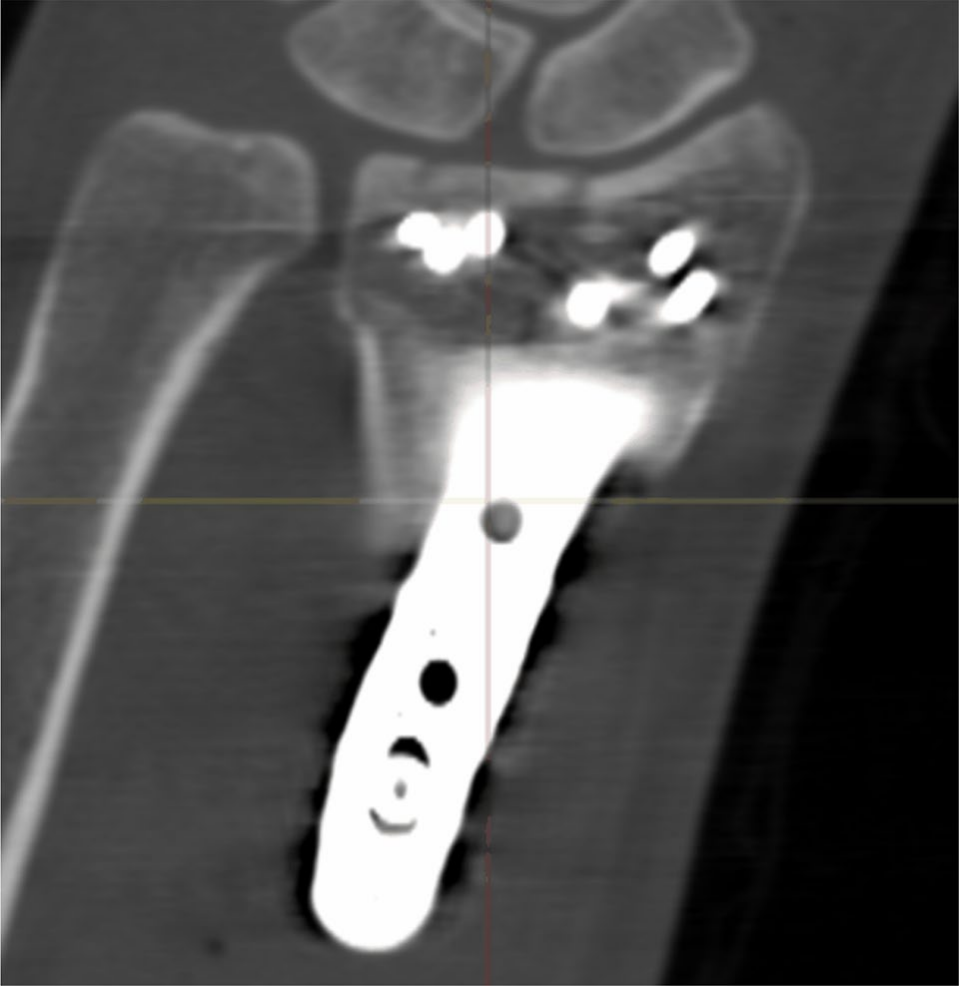
Postoperative scan of patient no. 3, with a < 1 mm gap in the articular surface

**Fig. 9:**
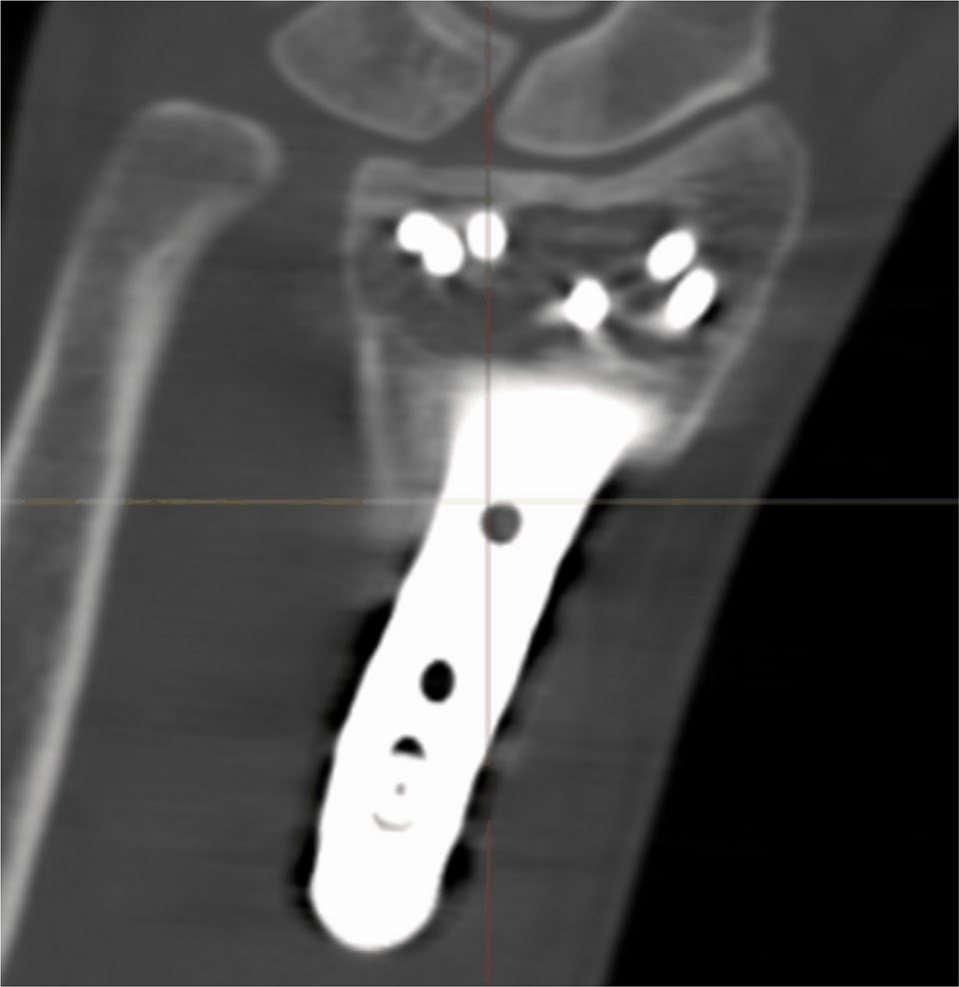
One-year follow-up of patient no. 3, with remodulation of the articular surface

**Fig. 10:**
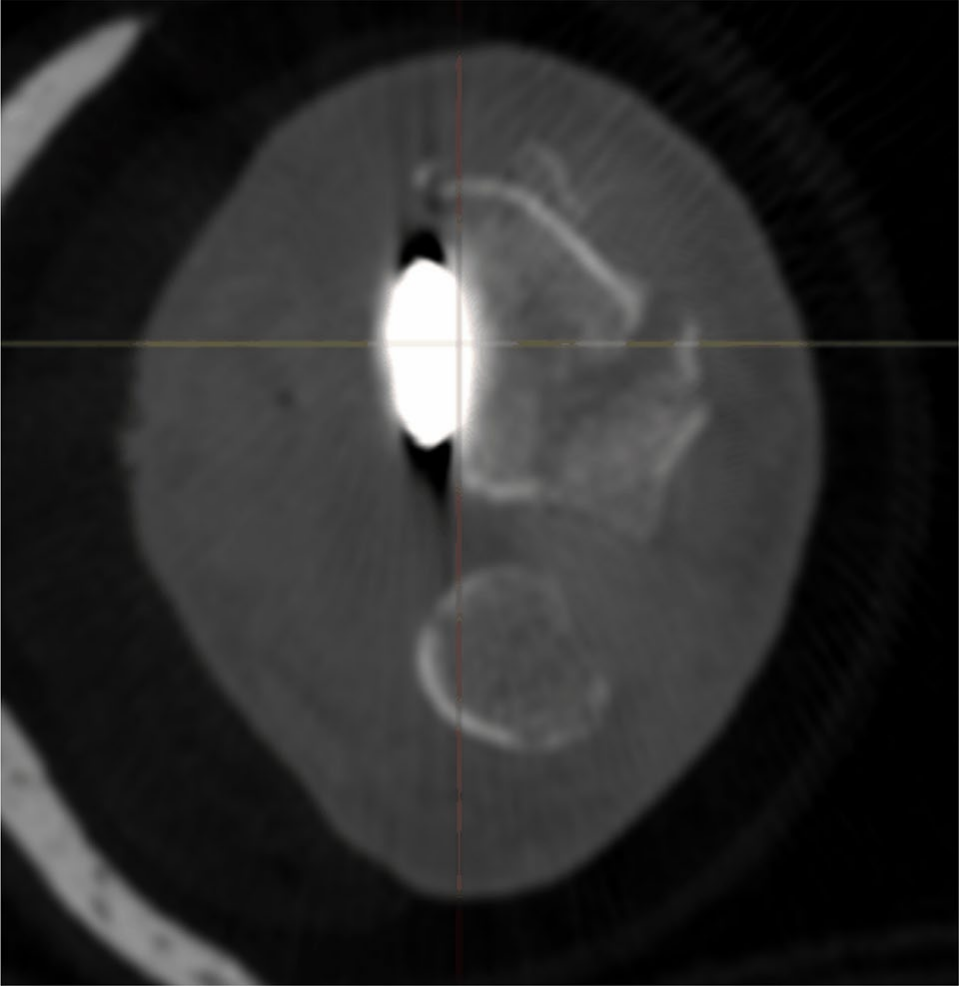
Postoperative scan of patient no. 6, with DUC fragment outlined

**Fig. 11:**
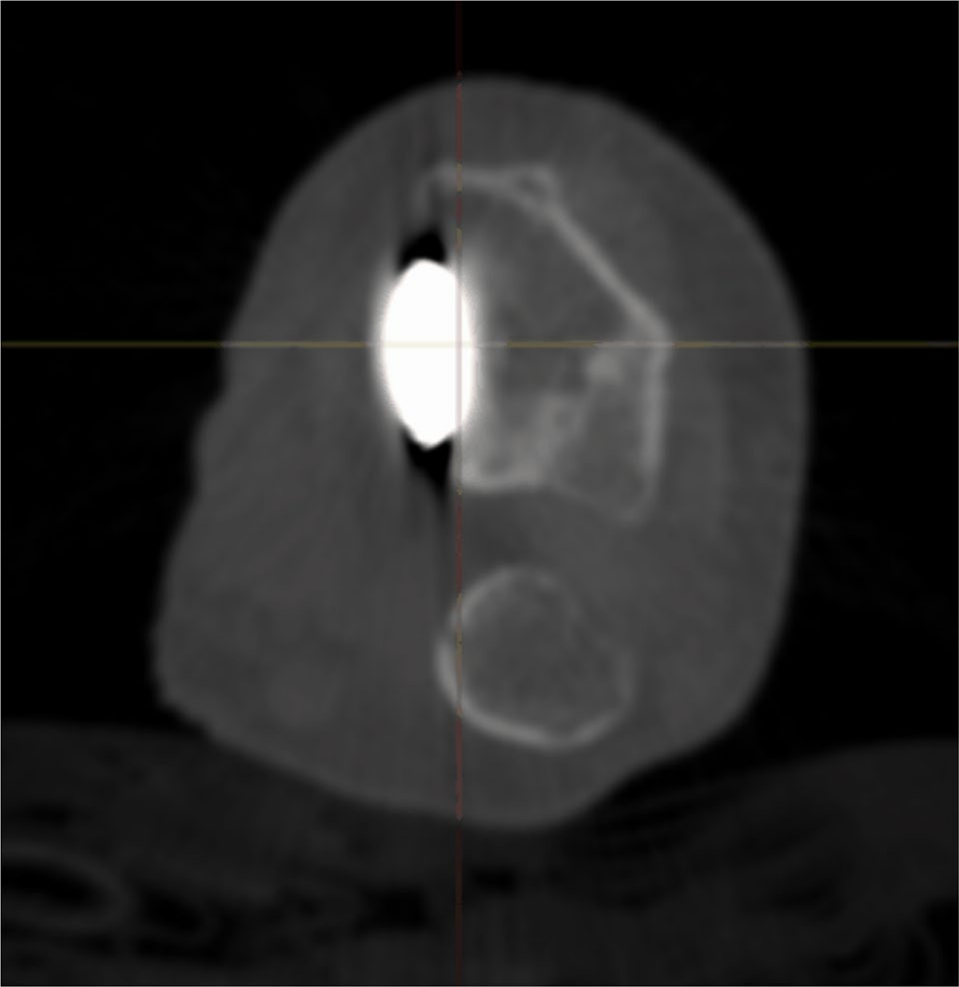
One-year follow-up of patient no. 6, with no migration/dislocation of the DUC fragment

Radiographic results are presented in Table 4. The median postoperative Batra score was 88 (range: 64–100). Seven of the 10 cases had a good to excellent Batra score. There was no case with articular incongruence > 1 mm postoperatively. No fragment migration was detected at the one-year follow-up, except for one case (no. 8) with increased dorsal tilt. A DUC fragment was found in 8/10 cases, fixated in 7/8, and not dislocated in any of the cases at the 1-year follow-up. The DUC fragment was fixated with 1–3 screws in each fragment. The median screw length was 82.6% (range: 64.2–126.5%) of the depth (distance between volar and dorsal cortex) of the distal radius. Of the screws in the DUC-fragments, there was one case (no. 8) with dorsal screw penetration. (Figs. 4, 5, 6, 7, 8, 9, 10, 11).

**Table 4:** Radiographic results

Patient no	Postoperative articular incongruence, > 1 mm	Maintained reduction at 1 year	Screw migration > 0–0.2 mm at 1 year	DUC fragment present	Number of screws in DUC fragment	Screw length, % of the depth of the distal radius		
						Screw 1	Screw 2	Screw 3
1	No	Yes	No	Yes	2	98.9%	85.6%	
2	No	Yes	No	Yes	3	73.7%	64.2%	70.6%
3	No	Yes	No	Yes	2	89.0%	81.4%	
4	No	Yes	No	Yes	1	71.0%		
5	No	Yes	No	No				
6	No	Yes	No	Yes	2	71.3%	90.0%	89.3%
7	No	Yes	No	Yes	0			
8	No	No	0.35– > 1 mm	Yes	1	126.5%	74.5%	
9	No	Yes	No	No				
10	No	Yes	0.2–0.35 mm	Yes	2	91.2%	82.6%

### Complications

There was no mechanical failure resulting in secondary surgery. There was no patient with load restrictions after the 3-month follow-up, no postoperative infections, and no cases of tendon rupture or complex regional pain syndrome. Plate removal was performed in one patient due to suspicion of intra-articular screw penetration.

## Discussion

In this study, we confirmed the usability of CTMA in a clinical setting and were able to distinguish motion in the injured part of the distal radius over time in patients operated for a DRF. The postoperative reposition was maintained and there was no fragment migration at the 1-year follow-up, except for one case with increased dorsal tilt. The DUC fragment was not displaced in any of the cases at the 1-year follow-up. Our findings, using a volume registration technique, indicate that a volar locking plate can yield and maintain a stable fixation of the fracture fragments including the DUC fragment.

This study included 10 cases, as it was a pilot study using CTMA for assessing radiographic outcome after DRF. The number of patients is a limitation and the results, therefore, may not be generalized.

In the present study, 8/10 patients with AO type C DRF had a DUC fragment; this is in line with previous studies showing that the DUC is involved in 87% of all AO type C3 fractures [10]. Bain et al. [25] demonstrated that the DUC is frequently involved in intra-articular DRF as one of the main sites when studying fracture pattern. Miyashima et al. found the mean size of the fragment to be 9 × 8 × 11 mm, occupying 50% of the DRU joint [10]. The DUC fragment can be challenging to reduce and capture because of its size and dorsal location. In the present study, the DUC fragment was fixated in 7/8 cases, with 1–3 screws in each DUC fragment, and the median screw length was 82.6% of the depth of the distal radius. In a recent randomized study including 150 patients with AO type C DRF treated with either volar locking plate or combined plating, the radiographic results were similar between the treatment groups and there was no case of re-displacement or mechanical failure after surgery [6].

In the present study, one of the 10 patients underwent hardware removal before the 1-year follow-up, due to suspicion of intra-articular screw penetration at the postoperative CT scan. Analysis of screw length regarding the DUC fragment showed that 1 of the 15 screws through the DUC fragment had penetrated the dorsal cortex. The subchondral screws are also important to support the articular surface after DRF. A biomechanical study concluded that locked unicortical distal screws of at least 75% of the length of the bone width can produce construct stiffness similar to bicortical fixation in extra-articular DRF, and at the same time avoid extensor tendon injuries [26]. This is not the case in intra-articular DRF with dorsal fragments or dorsal comminution. The DUC fragment is often small, and if the screws are too short, the fragment will not be captured. Too short screws will not give adequate fixation, and too-long screws mean dorsal prominence and penetration with risk of extensor tendon injury [27]. Ohno et al. [28] found that even when downsizing subchondral screws by 2 mm to prevent dorsal screw penetration, 9.6% of the patients still showed penetration during surgery or at the final follow-up. Detection of screw prominence and penetration is crucial, but difficult in the operating room. Conventional anteroposterior and lateral views are not sufficient to detect penetration [27], and so additional or alternative views and modalities are needed. Our findings show that the DUC fragment remained stable when stabilized by a single locking screw, and in one case with no locking screw. The reason for this is not entirely clear, but one explanation may be that stabilizing all the structures surrounding the DUC provides sufficient stability.

Movement of the distal screws in relation to the plate was minimal in 8/10 patients (< 0.2 mm) and more than 0.2 mm in the remaining two. Loosening of the polyaxial locking interface may result in loss of reduction, which was the case in one of these two patients (dorsal tilt). The strength of the locking interface of the variable-angle locking plate differs between implants, and an increase in the screw locking angle causes a reduction of strength which depends on the implant [29]. In our case, the loosening of the distal screws may have resulted from a technical error, such as incomplete locking of the screws to the plate.

This study showed no cases with incongruency in the articular surface on the postoperative radiographs, which is encouraging. Articular incongruence, with step or gap, predicts posttraumatic arthritis (PA), but the association between PA and PROMs is still unclear. However, wrist ROM is negatively affected by PA [30]. In this study, the clinical and functional results regarding pain, PROMS, wrist ROM, and grip strength were comparable to previous studies regarding AO type C DRF fixated with volar locking plate with 1-year follow-up [6].

As a next step, a larger cohort of patients treated surgically for a DRF would be needed to follow with CTMA for a longer time period to assess a relationship between fracture fragment movement and clinical outcomes. Given that the correlation between radiographic outcome and clinical outcome after a DRF is poor, it can be debated whether DRF patients benefit from radiographic follow-up. Nevertheless, AO type C DRFs represent the most complex fractures in the large group of patients. We believe these patients need optimal follow-up to facilitate postoperative rehabilitation. To our knowledge, the method of CT volume registration has not been used for DRF before; however, it seems to be suitable for assessing fragment size and migration, especially of key fragments of biomechanical importance to the wrist.

In conclusion, the findings in this study suggest that a variable volar locking plate can yield and maintain a stable reduction and fixation of the fracture fragments after AO type C DRF, including the DUC fragment. Further studies are warranted to determine the role and clinical significance of the DUC fragment. CTMA can be a valuable tool in the assessment of intra-articular DRFs.

## Data Availability

The data that support the findings of this study are available from the corresponding author, EL, upon reasonable request.
